# Authority and medical expertise: Arthur Conan Doyle in *The Idler*

**DOI:** 10.1136/medhum-2022-012491

**Published:** 2023-05-04

**Authors:** Anne Chapman

**Affiliations:** Glasgow Caledonian University London, London, UK

**Keywords:** Medical humanities, literature and medicine, cultural history

## Abstract

Arthur Conan Doyle’s medical and writing careers intertwined and his work has a history of being read in the light of his medical expertise. He wrote at a time when the professionalisation and specialisation of medicine had resulted in an increasing distance between the profession and the public, yet general practitioners relied financially on maintaining good relationships with their patients and popular medical journalism proliferated. A variety of contrasting voices often disseminated narratives of medical science. These conflicting developments raised questions of authority and expertise in relation to the construction of medicine in the popular imagination: how is knowledge constructed? Who should disseminate it? How and by whom is authority conferred? How can the general population judge experts in medical science? These are questions explored more widely in Conan Doyle’s writing as he examines the relationship between expertise and authority. In the early 1890s, Conan Doyle wrote for the popular, mass-market periodical *The Idler: An Illustrated Magazine*. His contributions to it address these questions of authority and expertise for a lay audience. First establishing the medical context of doctor/patient relationships in which these questions arose, this article undertakes a close reading of these mostly rarely studied single-issue stories and articles as a means of ascertaining how Conan Doyle and his illustrators identified the relationship between competing narratives, expertise and authority. It argues that rather than maintaining a distance between public and professional, Conan Doyle’s illustrated work demonstrates to his readers that there are ways to successfully navigate the appearance of authority and recognise expertise as they confront entangled representations of advances in medical science.

## Introduction

When tubercular patients travelled across Europe to Berlin hoping to access Dr Robert Koch’s experimental treatment, the scientific details of the remedy were being deliberately kept secret. An anonymous writer in WT Stead’s *Review of Reviews* condemned this concealment: ‘According to the rule of the profession, no cures wrought by secret remedies can ever be examined into. All dealers in secret remedies are quacks’ (Anonymous 1890, 547). In spite of this convention, the profession expressed intense interest in the secret cure. Koch had no intention of making details of his discovery public while the remedy’s efficacy was yet to be proven, but ‘exaggerated’ and ‘distorted’ reports forced him to provide some account (although he still did not reveal the origins and preparation of the remedy) (Koch 1890, 557). However, Koch’s colleague, Professor Von Bergmann, was to lecture on the subject in November 1890 in Berlin. At the time a general practitioner, Arthur Conan Doyle set off to Germany to seek the evidence for himself. During this trip, he failed to gain admission to Von Bergmann’s presentation or meet Koch, but did access patients receiving the treatment and discussed the lecture with others present. One result was a character sketch produced for the *Review of Reviews* expressing admiration for Koch, ‘the noblest German of them all’ (Conan Doyle 1890, 556). This episode in Koch’s and Conan Doyle’s careers highlights a conflict between expertise and authority in narratives of medical science. Political pressures forced Koch to announce the cure before he was ready; he was still developing his expertise away from the public. Once the cure was made public, the press, including Conan Doyle, constructed Koch as a hero. As Laura Otis puts it, ‘the voice of central authority could outweigh scientific evidence’ (Otis 1999, 25). This was a political central voice constructing narratives of national and imperial authority through a sympathetic press.

Nonetheless, a feature of fin-de-siècle mass-market periodicals was ‘to hold apparently contradictory discourses in suspension’ (Tattersdill 2016, 19) and, when we examine Conan Doyle’s presentation of Koch in the *Review of Reviews*, we find a variety of contrasting voices. The sketch is sandwiched between the aforementioned anonymous reflection on Koch (which connects his work to that of infamous quack Count Mattei)1 and Koch’s own defence of his decision to maintain secrecy around his discovery. One piece anonymously suggests potential quackery due to absent evidence, one portrays a heroic endeavour without having observed the hero, and one allows the expert himself to speak but he chooses to stay silent about the important details. Together these pieces do not comprise a definitive or cohesive presentation of Koch and his cure; rather, they leave the reader to draw their own conclusions based on evidence that has a range of obvious limitations. This mulitvocal, contradictory presentation of a key concern of medical science raises questions of authority and expertise in relation to the construction of medicine in the popular imagination: how is knowledge constructed? Who should disseminate it? How and by whom is authority conferred? How can the general population judge experts in medical science? These are questions explored more widely in Conan Doyle’s writing as he examines the relationship between expertise and authority; those with expertise do not always have authority in Conan Doyle’s narratives, nor does authority (understood as the power to influence) always coincide with expertise.

Researchers have examined the significance of the Koch episode for Conan Doyle and his contribution to popular understandings of medical science. Douglas Kerr argues that this was a key event in Conan Doyle’s writing career; he was ‘a writer in pursuit of a story’ who achieved a ‘journalistic scoop’ that would be his first piece for a national newspaper (Kerr 2010, 47, 46). In also reporting this story in the *Review of Reviews*, he contributed to the development of the martial metaphor as a way of comprehending the threats of the microscopic world, as Servitje (2021, 169–72) details. Emilie Taylor-Pirie (2022) identifies the event as a catalyst for Conan Doyle’s ‘passion for the narrative romance of medicine’ (144) and finds he positions ‘scientific brilliance’ opposing ‘microscopic threat’ (148). These readings recognise an admiration for the heroic Koch in this battle with bacteria. They also build on Laura Otis’ depiction of this opposition as having an affinity with Conan Doyle’s Sherlock Holmes stories, narratives of empire concerned with smallness as the hero detective confronts threats to the national body (Otis 1999, 96–100).

Conan Doyle’s role in a wider narrative of scientific heroism and empire is, then, well documented, predicated on the overlap between medical science and the consulting detective Holmes. There are, however, other facets to his shaping medicine in the popular imagination found elsewhere in his oeuvre, not least because his medical and writing careers intertwined. In his own time his work was read in the light of his medical expertise. A ‘Pen Portrait’ of him in the *Windsor Magazine* claimed that ‘his medical experience has been of the utmost value to him’ (Cromwell 1896, 367). In the same issue, MacLauchlan (1896, 369) details the effects of Conan Doyle’s medical training on his authorship claiming that, ‘[t]he scientific touch must in some degree colour all his work.’ (MacLauchlan 1896, 371). Conan Doyle himself would later, in *Memories and Adventures*, explain that his serial *The Stark Munro Letters* drew on his early years in medical practice, and that, famously, his tutor when he studied medicine, Joseph Bell, inspired the character of Sherlock Holmes (Conan Doyle, 1924, 52, 69).

In addition to the studies related to Koch detailed above, further readings of Conan Doyle’s work evidence arguments about nineteenth-century medical professionals. Peterson (1978, 93–7) reads *The Stark Munro Letters* as representative of the experience of a career in general practice; Furst (1998) uses some of Conan Doyle’s stories, including ‘The Doctors of Hoyland’ (122-3) discussed below, to evidence the changing power dynamics in the doctor/patient relationship; and Kerr (2013, 41–78) is interested in Conan Doyle’s presentation of divisions within the medical profession, in the way he contrasts consultant and generalist in order to investigate knowledge and the role of the expert itself.2 Conan Doyle’s work is also understood to have mediated Victorian cultural conceptions of the practice of medicine. For Moulds (2021), medical fiction played a part in the development of different professional medical identities and she demonstrates how a variety of Conan Doyle’s stories contributed to the emergence of those subdisciplines. However, his work not only represented late-nineteenth-century medical practice. The Koch episode suggests a new avenue of investigation in the relationship between his work and medicine: entangled in the inclusive and diverse contents of popular periodicals,3 his writing encouraged his reader to interrogate the construction of narratives around medical science through presentations of expertise and authority.

His hagiographical presentation of Koch shifted over time (Kerr 2010, 47). By 1924 he wrote more critically of the scientist and with more empathy for the public who had pinned such hopes on his cure. Kerr views this shift as influenced, in part at least, by Conan Doyle’s postwar opinion of Germany. But Conan Doyle’s contempt for ‘scientific arrogance’ (Kerr 2010, 47) and sympathy for a public who learnt of medical advancements through popular media can be found much earlier than 1924: between 1892 and 1894 Conan Doyle published seven short pieces in *The Idler: an illustrated magazine*, a periodical which actively positioned its readers in close relationship with its contributors. Proximity between the public and experts had significance in medicine during this period: it was a contradictory time in the practice of medicine when professionalisation and specialisation had resulted in an increasing distance between the profession and the public, yet general practitioners’ financial success depended on maintaining good relationships with their patients and popular medical journalism proliferated. It is unsurprising then that questions of authority and expertise were pertinent. Like the three pieces on Koch in the *Review of Reviews*, Conan Doyle’s *Idler* publications both raise and illuminate such questions. They comprise four stories that would later appear in Conan Doyle’s collection of medical fiction, *Round the Red Lamp* (‘The Los Amigos Fiasco’ (Conan Doyle, 1892c), ‘The Case of Lady Sannox’ (Conan Doyle, 1894a), ‘Sweethearts’ (Conan Doyle, 1894b) and ‘The Doctors of Hoyland’ (Conan Doyle, 1894c)), another story with a medical theme (‘De Profundis’ (Conan Doyle, 1892a)) and two non-fiction pieces (‘The Glamour of the Arctic’ (Conan Doyle, 1892b) and ‘My First Book: VI.—Juvenilia’ (Conan Doyle, 1893)).4

There is a critical precedent for reading these pieces together in their periodical context. Jonathan Cranfield’s study of Conan Doyle and the *Strand Magazine* (the enormously successful magazine that published his Sherlock Holmes short stories) emphasises the affordances of reading his fiction in its periodical contexts: recovering forgotten texts, and establishing a ’symbiotic’ relationship between fiction and non-fiction (Cranfield 2016, 2–3). Here I explore texts that have received far less critical attention than the Holmes stories, and bring two of his better known medical tales (‘The Case of Lady Sannox’ and ‘The Doctors of Hoyland’) into dialogue with rarely discussed pieces, a dialogue that would have been attended to by regular readers of the *Idler*. My analysis of these texts in relationship with each other affords a new insight into the author’s contribution to popular understandings of medical authority and expertise. Rather than emphasising a distance between public and professional, Conan Doyle’s illustrated work demonstrates to his readers that there are ways to successfully navigate the appearance of authority and recognise expertise as they confront entangled representations of advances in medical science.

## Recognising medical expertise and authority

Questions of authority and expertise are evident in developments in the medical profession towards the end of the nineteenth century, in particular those developments affecting all manner of professional relationships. The professional structure of medicine crystalised during the latter part of the nineteenth century. Following the Medical Act of 1858, which created the General Medical Council and initiated the registration of qualified medical practitioners, the separate groups of physicians, surgeons and apothecaries further cohered as medicine evolved from a dependent occupation to a profession with its own authority towards the turn of the century. The development most pertinent to my discussion was the changing relationship between the medical practitioners and their patients as medicine evolved. There was a distancing of medical practice from associations with trade, although many starting out in medicine found that economics ‘was nevertheless the bottom line in professional survival’ (Digby 1999, 4); there was also an emphasis on demonstrative professional courtesy and an attendant retreat of medical self-criticism from the public eye and thus public judgement (Peterson 1978, 250–55). Yet medical practitioners depended financially on maintaining good relationships with the public and this public was nonetheless interested in and familiar with medical developments. These relationships were complex, often mediated by the press. Frampton (2020) details the development of nineteenth-century medical periodicals and finds that the end of the century saw both specialised publications (by their nature distanced from laymen) and journals that aimed to ‘mak[e] medical knowledge accessible and interesting to lay audiences’ (Frampton 2020, 450). She explains that this worried established publications: the *Lancet* for example, raised the dangers of self-diagnosis (Frampton 2020, 450). But the *Lancet* itself was a journal that set out to make medical knowledge accessible, which suggests a conflict around who has the authority to distribute medical knowledge to laypeople and the effects of this distribution. Adopting new therapeutics that had been enthusiastically discussed in the press could effect public approval and accompanying success for the doctor using them: for example practitioners who made use of cocaine’s anaesthetic qualities to treat patients absorbed some of the prestige surrounding the drug in the late nineteenth century (Small 2016). This in turn garnered a more general public perception of practitioners’ heroism and ‘the physician’s unassailable moral primacy’ (Small 2016, 4). Patients developed conflicting expectations of the doctor/patient relationship, demanding both the latest treatments and the comfort of more traditional practice (Furst 1998, 179).

Successful doctor/patient relationships depended not only on treatment and the latest medical expertise, however. ‘Observing medical etiquette was conceived as an important route to achieving professional acceptance and success, particularly for those who lacked social contacts or capital’ (Moulds 2019, 104). Etiquette guidance for doctors recognised that conduct towards patients could disguise other deficiencies too: ‘if one is especially polished and elegant in manner, and moderately well-versed in medicine, his politeness will do him a great deal more good with many than the most profound acquaintance with histology, microscopic pathology and other scientific acquirements’ (Cathell 1889, 42). More than once this guide advises that politeness and sensitivity impress the patient more than medical knowledge, and emphasises the importance of patients’ perception and expectation of events. For example, it states that the physician should ‘not make your visit so brief or abrupt as to leave the patient *feeling* that you have not given his case the necessary attention’ (Cathell 1889, 46, my emphasis). What is important is the patient’s own perception of requirements, rather than the medical requirements of the case, unsurprising when ‘[y]our professional fame is your chief capital’ (Cathell 1889, 39). This advice encouraged doctors to perform an expected appearance of authority, an authority that could have little to do with medical expertise.

These developments that made establishing the veracity of medical authority difficult for patients had potentially serious consequences. One problematic symbol exemplifies this. In the 1880s and 1890s, the red lamp, hung to advertise medical services, also symbolised the potential disjuncture between the appearance of authority and the reality of expertise and the difficulty of distinguishing between the two. Understanding what was at stake in this symbol reveals the significance of Conan Doyle’s writing in the *Idler*. ‘Anybody […] is at liberty to hang a red lamp over his door and […] to sell drugs and poison without restriction, and to “make up” prescriptions with impunity’ (Anonymous 1883, 642). With little other recommendation than the lamp, those seeking urgent care could find themselves receiving treatment from someone entirely unqualified to provide it. This was the case in 1882 when a child died due to misdiagnosis by a doctor’s assistant; the child’s parents believed him to be a doctor due to the red lamp outside his house (Anonymous 1882, 694). Following an 1894 inquest, a contributor to the *British Medical Journal* summarises the issue thus:

In framing any attempt to suppress quackery the fact must be recognised that great numbers of the masses are quite willing to accept the red lamp as sufficient, while many of the classes, it is to be feared, take a certain sceptical pleasure in believing that non-qualification is at least one index of an open mind and a thing therefore rather worthy of encouragement. (Anonymous 1894, 1261)

When Conan Doyle (1894d) published his collection of medicine-related short stories, *Round the Red Lamp*, readers would have recognised the title’s associations with the disconnect between giving the appearance of authority and having the expertise to justify it. A reading of his medical stories in the *Idler*, however, recognises that concerns about and understandings of expertise and authority were part of wider cultural questions of knowledge creation, dissemination and consumption that nonetheless affected how the public related to medical practitioners.

## Conan Doyle and *The Idler*

While Conan Doyle framed *Round the Red Lamp* with a preface and final story that suggest a reading that emphasises a distinction between expert medical practitioner and general reader (a likely patient), the publication context of the *Idler* inflects his medical stories differently, bringing the public and practitioner into close proximity imaginatively. In the *Idler* the stories overlap with other contexts and particularly with the idea of the gentlemen’s club.

The *Idler* first appeared in February 1892 under the editorship of Jerome K Jerome and Robert Barr (Dunlap 1984, 178). It was announced with some fanfare: the *Review of Reviews* described it as a ‘formidable rival’ for the *Strand Magazine* (Anonymous 1892, 188). Published circulation figures suggest its popularity,5 as does its persistence until 1911, although with slight changes to its title and with other editors (Dunlap 1984, 181–2). In the context of the *Idler,* illustration contributes to Conan Doyle’s narratives providing emphases not available in other contexts. A reading such as mine, which places significance on the periodical context in creating meaning, must necessarily attend to illustration. A range of scholarship suggests how we might read nineteenth-century illustrated texts. Patten (2002, 91) explains that ‘[i]llustrations are not mimetic. They are not the text pictured. […] To say, then, that an illustrator illustrates a text might mean that the artist enlightens the text’. In other words, they enhance our understanding of the narrative, rather than merely represent it. Sillars (1995, 17) explains how technological developments in print ‘created a unified discourse of word and image’, thus suggesting we read an illustrated text as a whole, all parts contributing to its meaning. And Julia Thomas has demonstrated the significance of reading these texts as unified discourse. She finds that ‘[m]eanings are generated in the very interaction between the textual and the visual, the points at which they coincide and conflict’ (Thomas 2004, 15). Illustrations work with the verbal text to make meaning, thus influencing our reading of Conan Doyle’s work in the context of the *Idler*.

The *Idler* was a journal comfortable with being seen to meet mass-market demand at a time when the New Journalism was elsewhere stigmatised (Fiss 2016, 418). Indeed, it featured a column in which its contributors ‘treat[ed] the notion of exclusivity itself as a joke’ (Fiss 2016, 420). This serial column was known as ‘The Idler’s Club’. Laura Kasson Fiss’s study of it explains that ‘[m]agazine readership is commonly associated with mass consumerism, commercial transaction, and anonymous global distribution, whereas the club suggests a cozy friendship among select members that excludes everyone else’ (415). Fiss finds the tone of the club column to foster a relaxed atmosphere in which the reader has some intimacy with literary celebrities; it ‘imitated club talk’ (420). Thus this column created ‘an intimate imagined space for its mass readership’ (415). If we are to read the column as Fiss suggests, as a ‘microcosm of the journal as a whole’ (426), then such an imagined space inflects the contents of the *Idler* with an intimacy that brings Conan Doyle’s readers close to him when he publishes there and positions his work as already resistant to distance between professionals and laypeople. Rather than produce narratives emphasising an unassailable distinction between the medical practitioner and the layman, his writing in the *Idler* positions readers where they can consider what it means to be on the brink of the unknown and how knowledge is created. In this context, Conan Doyle’s writing encourages his readers to challenge appearances of authority in medical practice, and to recognise instead the significance of expertise, with its dependence on experience and effort.

## De Profundis

‘De Profundis’ illustrates the competing narratives of interpretation that can challenge expertise in medical science. Conan Doyle’s first piece in the *Idler* concerns illness, diagnosis and death (Conan Doyle, 1892a). At first it seems to be a straightforward tale that suggests the folly of resisting professional help. The story features John Vansittart and his wife, and is narrated by his agent. The couple are due to sail to Columbo when Vansittart becomes ill. When he first announces he is unwell, the narrator tells Vansittart that ‘a touch of the sea will set you right’ and he responds: ‘I want no other doctor’ (151). Vansittart’s behaviour and appearance suggest that he is drunk and indeed the narrator convinces himself of this stating ‘[s]ad it was to see so noble a young man in the grip of that most bestial of all the devils’ (151). Yet we soon discover the narrator’s misdiagnosis: Vansittart has ‘not had a drain for two days’ (152). Vansittart himself diagnoses London to be the cause and proposes being at sea as the cure. The narrator attempts to persuade Vansittart to see a medical doctor, but he refuses, preferring to trust in the presence of the ship’s surgeon to ensure his safe journey. Once aboard, Vansittart’s illness is diagnosed as smallpox and his subsequent death at sea implies the folly of self-diagnosis. Conan Doyle demonstrates the confident ease with which a layman will mistakenly interpret the evidence and the dangers of refusing to engage with experts. However, the story’s conclusion complicates this.

Anticipating the conclusion, the narrator’s introduction imagines a mother ‘seeing’ her dying son and he states: ‘Far be it from me to say that there lies no such power within us’ (149). He acknowledges the possibility of the supernatural here, but firmly encourages scepticism: ‘once at least I have known that which was within the laws of nature to seem to be far upon the further side of them’ (149). The conclusion of Vansittart’s story illustrates his point: the significance of the title is that the body of Vansittart rises out of the depths in what appears at first to be a vision. The narrator and Vansittart’s wife are sailing to Madeira, thinking they will be able to meet up with and nurse the sick man there. When his body bursts out of the depths and sinks back below, and unaware that he has already died aboard ship, his wife believes she sees a vision signifying he has died at that very moment. When they catch up with the Captain of Vansittart’s ship, they find in fact he had died 8 days previously and been buried at sea. This knowledge changes the narrator’s interpretation of what they have seen; he concludes it must have been Vansittart’s actual body. The vision appeared at the exact place of burial, the surgeon says the weight was not well attached, and the noises could be explained by the presence of sharks. Nonetheless, ‘a clearer case of a wraith has seldom been made out, and since then it has been told as such, and put into print as such, and endorsed by a learned society as such’ (157). So, in the case of Vansittart’s illness, the narrator’s and Vansittart’s misdiagnosis suggests a need to rely on expert intervention, but in the case of Vansittart’s death, the narrator’s very plausible explanation drawing on a surgeon’s expert evidence contradicts that of other experts. What ‘De Profundis’ does then, is at once emphasise the need for informed interpretation but reminds the reader that experts may interpret differently, that there are competing narratives to navigate in order to understand the world.

## The Glamour of the Arctic

Conan Doyle’s second piece for the *Idler* explores the difficulties of navigating competing narratives in the pursuit of knowledge more expansively. Although only its conclusion addresses medical science, ‘The Glamour of the Arctic’ demonstrates the effect of combining multiple interpretations and expertise from different sources as they intersect with the popular imagination in the construction of knowledge. Such a scenario reflects the general reader’s encounters with medical science in the popular press, such as the Koch episode in the *Review of Reviews*. In 1880 Conan Doyle paused his medical studies to work as a surgeon on board the Arctic whaling ship *Hope*. Here he experienced an adventure he would describe in his autobiography as ‘a strange and fascinating chapter of [his] life’ (Conan Doyle, 1924, 41). Twelve years later, and four months after ‘De Profundis’, ‘The Glamour of the Arctic’ wove together scenes from life on a whaling ship and the whales they hunted (Conan Doyle, 1892b). Whaling was in decline and its practice altered from the way it was conceived in popular imagination. At the same time, the Arctic and its history were yet to be fully apprehended.

The text wavers between expertise, mystery and imagination. Conan Doyle describes a useless (in commercial terms) rorqual whale is ‘eighty foot’ but its spray is ‘like smoke’ and ‘where green is turning to black the huge flickering figure […] glid[es] under the ship’ (627). It is both measurable, but insubstantial; he uses the word ‘strange’ here to describe both the sight and sound of the whale, yet he is able to compare the gullet size of different types (627). The same occurs when he comes to the harpooner. He is quick to dispel any notions his readers may have gathered from books; he explains that this ‘gallant seaman, who stands at the prow of a boat waving harpoon over his head, with a line snaking out into the air behind him, is only to be found now in Paternoster Row’ (627). This is a figure relegated to print, and, it seems, for good reason. Quite simply, ‘one could shoot both harder and faster than one could throw’ (627-8). However, at the same time Conan Doyle wants to cling to the popular but outdated image, welcomes its outrageousness and impossibility in print at least. The glamour of the Arctic is not only a result of physically experiencing it, then; it is also effected imaginatively, through popular understandings perpetuated on the page. The popular imagination holds sway in the face of new expertise and mystery is stubbornly persistent even as factual understanding is attained.

This imbrication of knowledge and awe also occurs when he relates the whaler’s experience. The romance of the Arctic colours their expertise. Indeed, he notes that ‘[s]ingular incidents occur in those northern waters, and there are few old whalers who have not their queer yarn’ (635). His inclusion of anecdote and tall tales only adds to the hazy glamour with which this text veils the Arctic; he weaves imaginary understandings with the facts he presents. Making use of anecdote also affords him the opportunity to suggest the possibility of finding a passage to the North Pole, though he gives the caveat that ‘some little margin must be allowed, no doubt, for expansive talk over a pipe and a glass’ (633). He writes of ‘gnarled and rugged’ old ice, ‘impossible to pass’, and relates an 1827 attempt where it seemed the impassable ice persisted to the pole (634). Then he adds the whaler’s view that there have been times when they have seen no ice at a similar distance North to that 1827 voyage. These are differing experts’ tales and the reader is left to wonder what could be the truth of it .The accompanying illustrations add to the sense of competing views.

**Figure 1:**
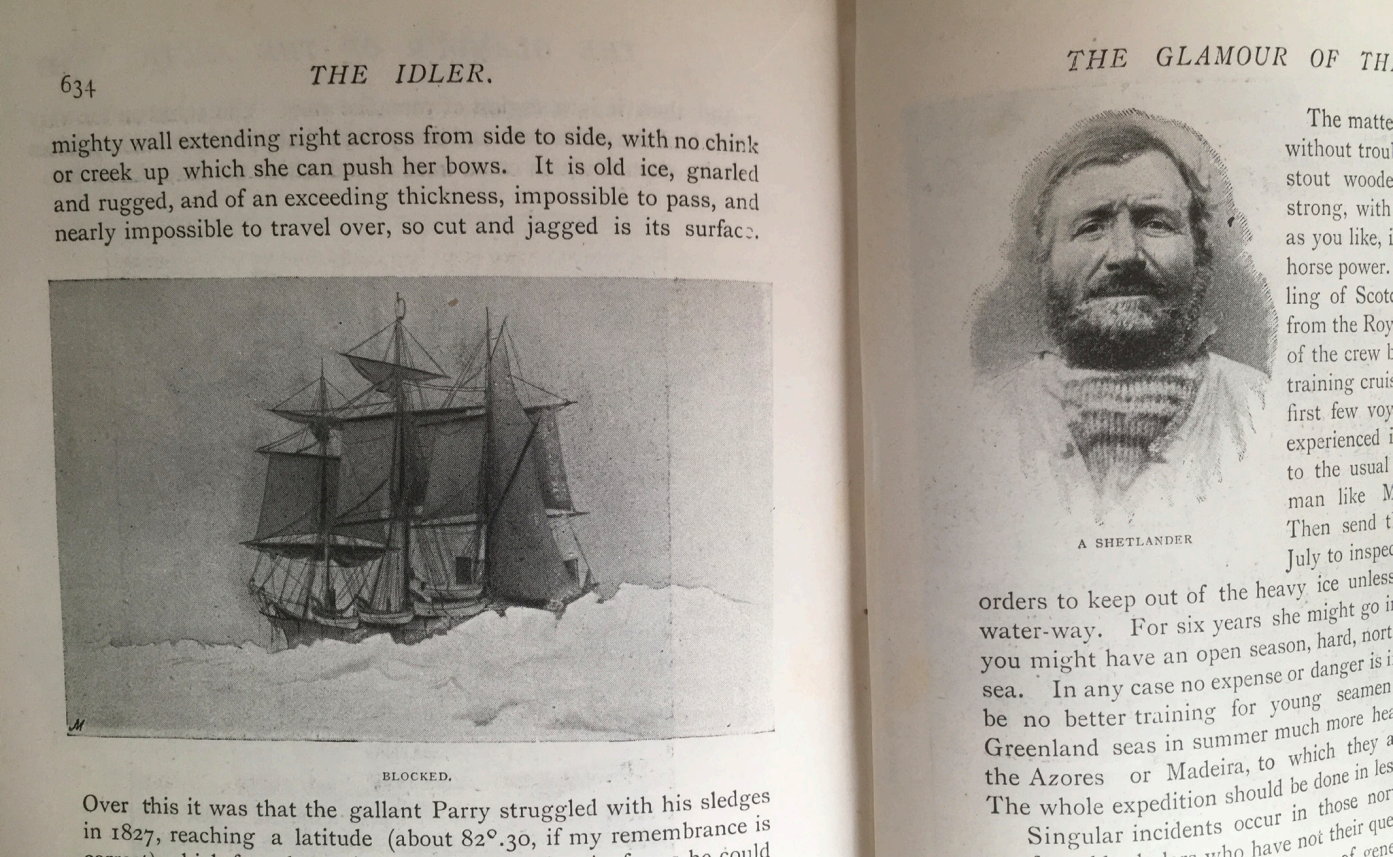
Double page from ‘Glamour of the Arctic’ (adapted from Conan Doyle, 1892b, 634–5) featuring the illustration ‘Blocked’ by A Webb and a photograph of ‘A Shetlander’.

‘Blocked’, an illustration by A Webb, intersects the different stories of the attainability of the North Pole (634. See figure 1). Here the ice obscures the ship and the crew are absent. Webb gives no hint of them among the rigging or on deck or attempting to man the boats. The edges of the drawing of the ship are sharp and straight, contributing to the impression of an absence of movement. The whalers’ experience is thus diminished. The stillness of the ship contrasts with the looming of the ice jaggedly rising in the foreground. Its dominance and its apparent thwarting of the ship’s progress make it difficult to imagine that the ice could clear. Thus the image casts at least some doubt on the whaler’s account of ice-free seas.

On the opposite page, however, the photograph of the Shetlander illustrates a suggestion made by Conan Doyle that the possibility of clear passage to the pole could be tested (635. See figure 1). He suggests that a crew comprising ‘Scotch and Shetland seamen from the Royal Navy' could make yearly expeditions with a chance of finding clear sea (635). This photograph is sharp, solid. This Shetlander, titled so as to appear representative of a type, looks the reader right in the eye, his mouth set firm. There is an appearance of authority in this image; it appears to be a reliable image of confidence and determination, strengthening the plausibility of Conan Doyle’s proposition. The reader is encouraged to judge authority from appearance.

These two images across the spread of the two pages have a similar effect to Conan Doyle’s narrative shifts. We move from an emphasis on impassable ice on one page to a type of man with the tenacity to overcome the growing obstacle on the next. The shifts between Webb’s illustrations and the photographs as they imbricate with the text both allow for the precision and romance that create ‘The Glamour of the Arctic’ and give a sense of the spell cast by a whaling voyage among icy seas. The illustrated text as a whole suggests what Conan Doyle might mean when he writes ‘You stand on the very brink of the unknown’ (633). The competing narratives attendant on the production of new knowledge can fail to provide clarity, but the desire to achieve it is intoxicating.

In contemplating that brink, Conan Doyle implies in his concluding paragraph that in writing the ‘The Glamour of the Arctic’ he has been dazzled. He states, ‘There is a good deal which I had meant to say’ as if somehow distracted from his purpose. He at once destabilises his authority to relate the things that ‘throw the glamour over the Arctic’ as he also acknowledges that these missing utterances have ‘all been said much better already’ (638). Nonetheless, his final move is to present himself as innovative in writing on the region, and he does so in a way that draws on his own area of expertise: he attends to the Arctic’s ‘medical and curative side’. The haze seems to lift: ‘Davos Platz has shown what cold can do in consumption’ and ‘in the life-giving air of the Arctic Circle no noxious germ can live’ (638). Straightforward and with clarity, Conan Doyle presents what he terms a ‘safe prophecy’: ‘before many years are past, steam yachts will turn to the North every summer, with a cargo of the weak-chested, and people will understand that Nature’s ice-house is a more healthy place than her vapour-bath’ (638). It seems an ideal grounded scientifically in the Arctic air, not in its enchanting glamour. It is an ideal seemingly far from maintaining the romance that so appealed to the author.

**Figure 2:**
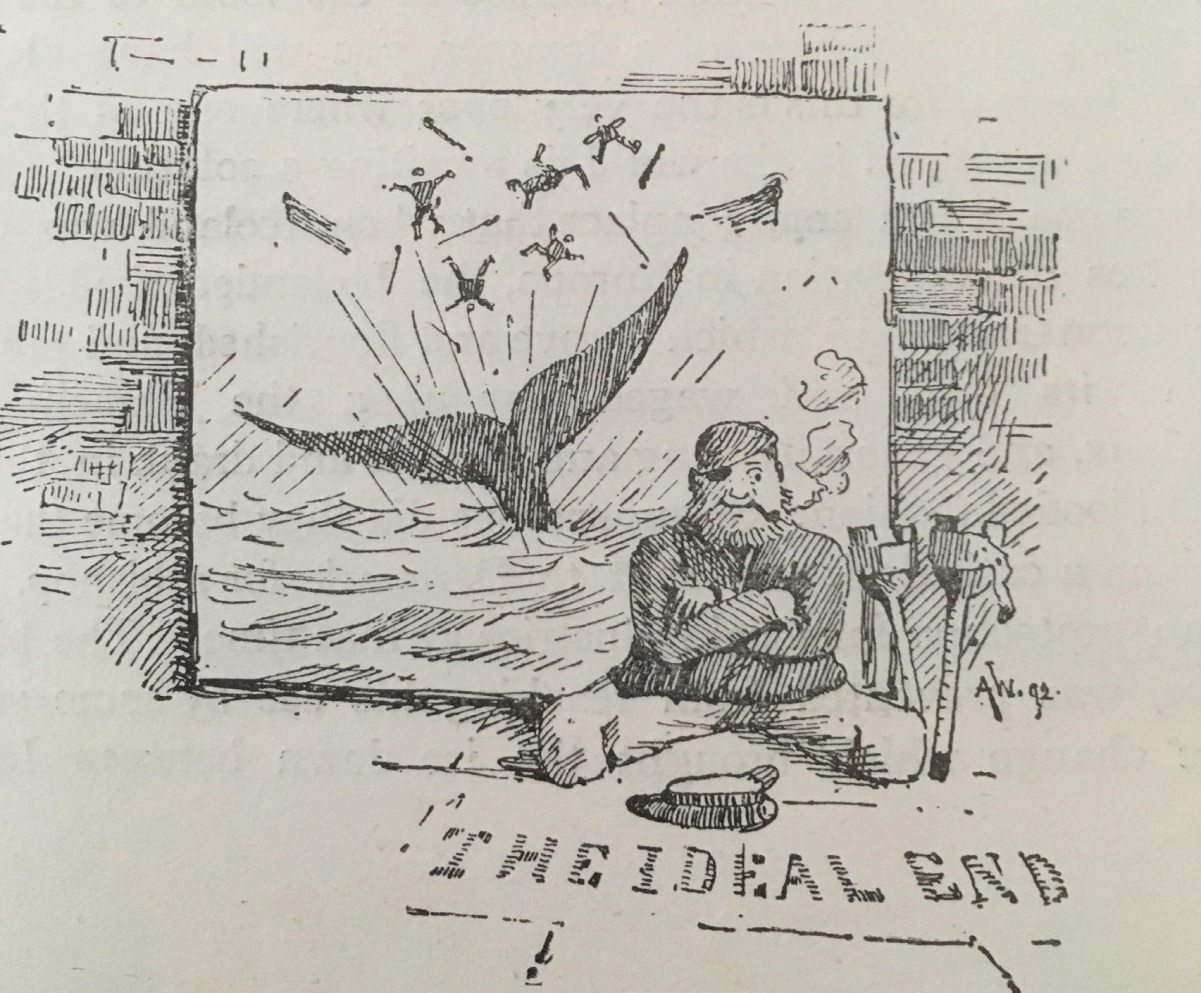
A Webb. ‘The Ideal End’ (adapted from Conan Doyle, 1892b, 638).

But Conan Doyle’s words are not the end of the narrative, rather Webb’s final illustration (638. See figure 2) brings us back to both the whale and the expert seamen with whom Conan Doyle was so in awe. This illustration differs greatly from Webb’s other images. Where they reflected the Arctic haze through soft washes, here Webb hatches lines. There is no haze in his visual representation of his ideal end. And so both Conan Doyle and Webb conclude with a clarity emerging from the haze of the rest of the piece. However, Webb seems utterly at odds with Conan Doyle. In bringing us back to the whale and its potential for devastation, his illustration seems to mock Conan Doyle’s idealisation of the medical benefits of an Arctic voyage where these creatures lie beneath the surface. The begging seaman, apparently injured by a whale, smiles nonetheless, perhaps encouraging us not to pity him but to laugh at Conan Doyle’s proposition given the danger. And yet the seaman’s illustration of his disaster returns us to Conan Doyle’s earlier description of harpooning a whale. He wrote: ‘should the whale cast its tail in the air, after the time-honoured fashion of the pictures, that boat would be in evil case, but, fortunately, when frightened or hurt it does no such thing, but curls its tail up underneath it, like a cowed dog, and sinks like a stone’ (628). If that is the case, then the seaman’s picture, the potential danger of whales, seems to be pure imagination. Conan Doyle’s prophecy of curative voyages can exist with the other ideal, the romantic notions of the whale, and of whaling.

The glamour evoked by Conan Doyle, Webb and whoever selected and set the photographs, layering experience and hearsay with the measurable and the elusive, suggests that expertise can sit comfortably with incomprehension and awe. ‘The Glamour of the Arctic’ portrays a value in imaginative, uncertain ways of comprehending the world, and Conan Doyle’s, and indeed the *Idler*’s editors’, authoritative weaving together of these very different types of expertise suggests the desirability of drawing on very different experts to make sense of experience.

## The Los Amigos fiasco

‘The Los Amigos Fiasco’ (Conan Doyle, 1892c) was listed separately from the list of medical stories in Conan Doyle’s accounts diary (Conan Doyle, 1892d). However, its subsequent inclusion in *Round the Red Lamp* suggests Conan Doyle considered it to be related to the issues of medical authority exemplified by the lamp of the title. Where ‘Glamour’ explores the difficulties of untangling competing expert and imaginative understandings, ‘Los Amigos’ contrasts expertise with unearned authority. It is certainly not a serious story; it concerns a failed attempt at execution by electrocution, which, far from killing the prisoner, seems to have made him invincible. The only medical presence in this tale is the surgeon attending the execution and the prisoner’s reference to a swathe of doctors puzzled by his troublesome arm (rendered ‘good as new’ by the electrocution). This humorous tale goes further than to suggest that medical expertise is finite; it positively encourages its readers to laugh at authority, in other words to discount its appearance of power, when it is unearned.

Significant to Conan Doyle’s purpose here are the aspects of the tale dealing with the refusal of those in authority to accept the opinion of an amateur expert. The narrator is a journalist John Murphy Stonehouse, who, in the interest of financial reward, sets out to ‘tell the true facts’ about the case (548). Duncan Warner is condemned to be the first to be executed via the powerful dynamos of Los Amigos and the town council have appointed four experts to oversee proceedings. Three of these experts appear to be considered so because of professional success. The fourth, Peter Stupnagel, was ‘regarded as a harmless crank who made science his hobby’ who never published his results, although Conan Doyle clearly implies his expertise, for ‘he was *eternally* working with wires and insulators and Leyden jars’ (550, my emphasis); in other words he is continually gaining experience of that about which he will later explain. Stupnagel sits quietly through the meeting where the group plan the execution, but as it draws to a close he contradicts their decision to sextuple the charge from the strength previously used in New York. He states, ‘Gentlemen […] you appear to me to show an extraordinary ignorance upon the subject of electricity. You have not mastered the first principles of its action upon any human being’ (551). He continues to question their ‘assumption’, asking ‘Do you know anything, by actual experiment, of the effects of powerful shocks?’ (551). Their ‘pompous’ response sets up Conan Doyle’s argument about what constitutes expertise: ‘We know it by analogy’ (551), in other words they lack experience. In the face of this presumption, Stupnagel offers evidence about the effect of electricity on the human body, which is duly ignored and the committee outvotes him. The incredible failure of Warner’s execution, rendering him apparently immortal, signifies that practical experience (in this case experiment) over authoritative position, is the reliable source of information. As the story concludes, a marshal declares to Stupnagel: ‘You seem to be the only person who knows anything’ (556). Stupnagel’s expert knowledge was what mattered, not the elevated professional positions of the others when that position was underpinned by ignorance. Conan Doyle suggests in this funny, but otherwise slight, story, that expertise can be achieved by anyone committed to developing it, and that those with authority may have unwarranted confidence in their own ability to theorise. Thus he provides his readers with a way to begin to untangle some of the competing narratives around medical science, like those depicted at the conclusion of ‘De Profundis’. Certainly, new knowledge must draw on expertise gained by experience and not theory alone.

## My First Book: VI.—Juvenilia

**Figure 3:**
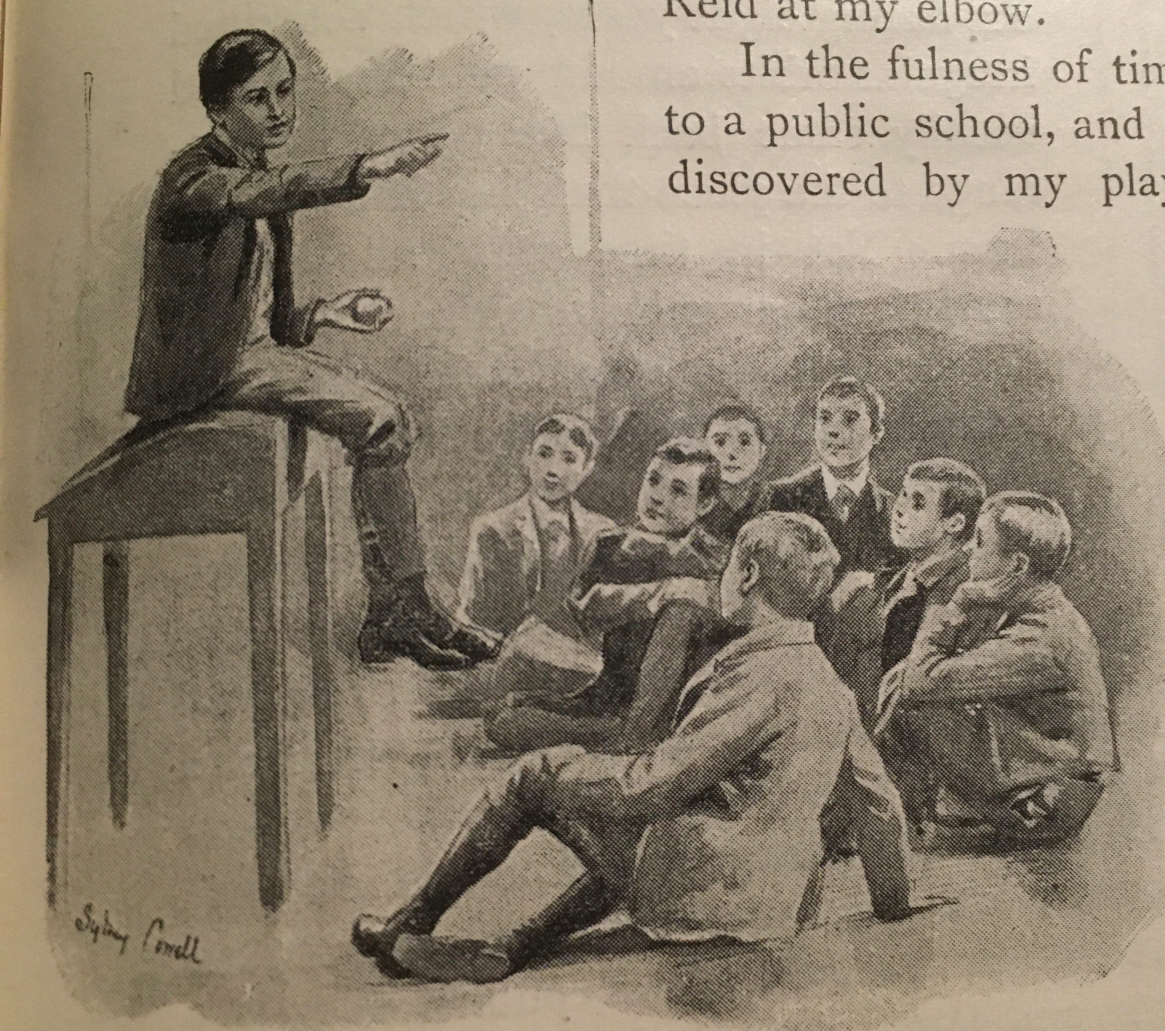
Sydney Cowell. ‘My Debut as a Story-Teller’ (adapted from Conan Doyle, 1893, 635).

If ‘Los Amigos’ contrasts expertise and authority, ‘My First Book’ connects them as Conan Doyle reflects on how he developed both, implicating the public in the production of professional authority. He explores the development of expertise (his own) in more depth and identifies the basis of authority to lie in public relation to others (Conan Doyle, 1893). This illustrated biography presents Conan Doyle as an authoritative story-teller from a young age. The illustrator, Sydney Cowell, depicts the schoolboy Conan Doyle sitting on a desk, ‘elevated’ as the author puts it, while smiling boys gaze up at him ‘little boys all squatting on the floor’ (635. See figure 3). Both Cowell and Conan Doyle thus make a distinction that confers authority on the story-teller. Furthermore, we learn that his school friends were willing to pay for his work: ‘I was bribed with pastry to continue these efforts, and remember that I always stipulated for tarts down and strict business, which shows that I was born to be a member of the Authors’ Society’ (635). Of course, there is an intentional humour here but nonetheless, in describing this public authoritative performance, Conan Doyle is constructing an image of a member of a profession. He implies that authority and professionalism occur in public. Like Stulpnagel, Conan Doyle’s expertise, however, is developed much more privately.

That development is facilitated through practice and application, in this case reading. Conan Doyle’s account describes the effect of reading as both conferring special knowledge and affording a visceral experience, even while he presents himself as exceptional. This in turn suggests his expertise as a story-teller. First, he describes years reading unusually intensely, joking about a rumour that ‘a special meeting of a library committee was held in [his] honour, at which a bylaw was passed that no subscriber should be permitted to change his book more than three times a day’. And he continues to explain that he ‘managed to enter [his] tenth year with a good deal in [his] head that [he] could never have learned in classrooms’ (634). Here then, Conan Doyle is presenting himself as a prodigious reader who has learnt more from his private reading than at school. This depiction suggests that a personal motivation in developing knowledge is most productive.

If that discussion of his development could seem perhaps arrogant, his discussion of learning his writer’s craft and his humorous loss of a manuscript reveals he clearly did not feel himself to be immediately expert: ‘Of course it was the best thing I ever wrote. Who ever lost a manuscript that wasn’t? But I must in all honesty confess that my shock at its disappearance would be as nothing to my horror if it were suddenly to appear again—in print. If one or two of my earlier efforts had also been lost in the post my conscience would have been the lighter’ (637). His humour is disarming; there is no arrogance here. With his openness about his failures, Conan Doyle portrays himself as possessing humility, but his persistence, and the use of the term apprenticeship, suggest a levity accompanied by determination. He may laugh, but no one could accuse him of not taking writing seriously. This is hard work.

**Figure 4:**
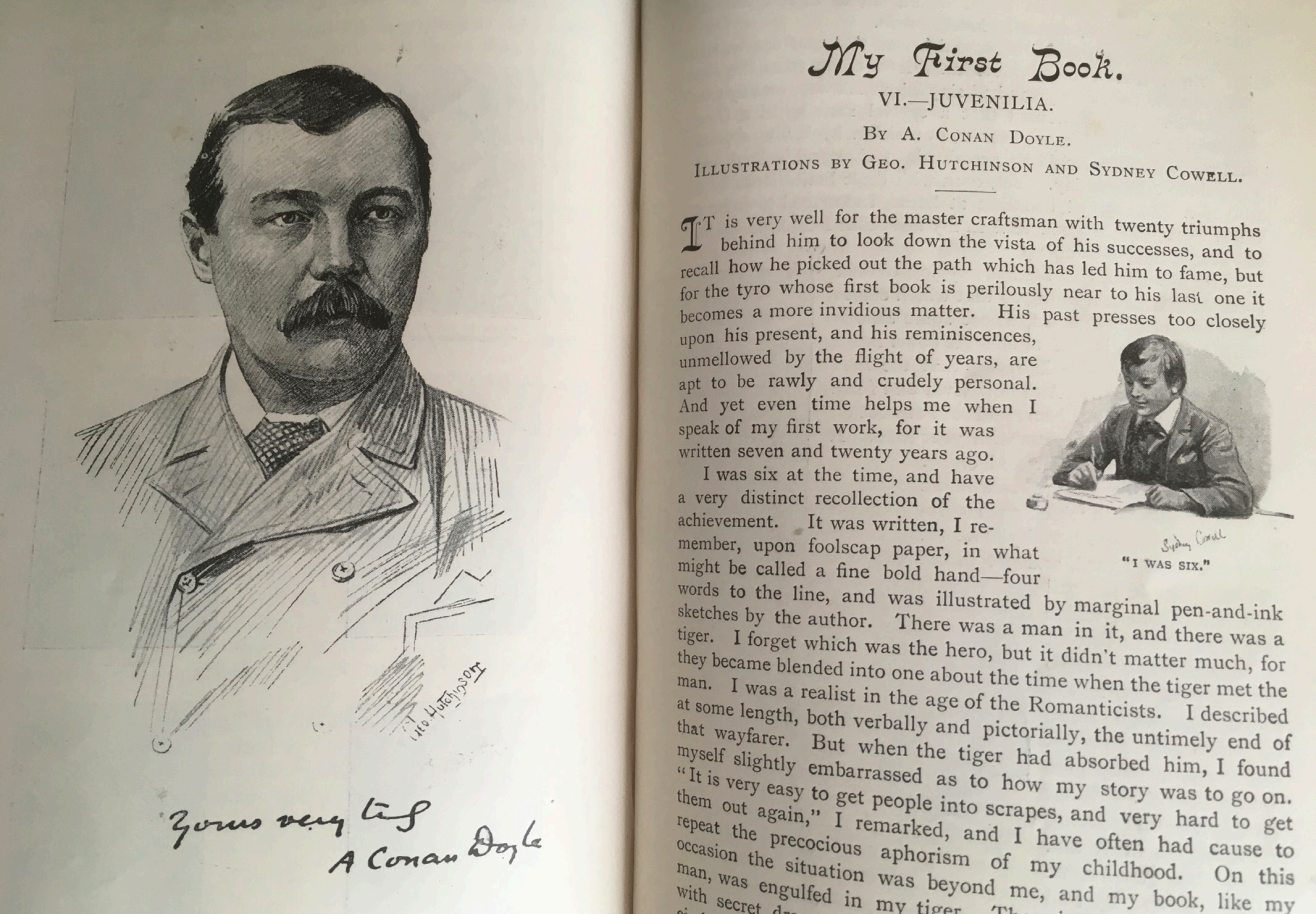
Double page of ‘My First Book-Juvenilia’ (adapted from Conan Doyle, 1893, 632–3) featuring a portrait of Conan Doyle by George Hutchinson and Sydney Cowell ‘I was Six’.

This focus on hard work and persistence from the outset suggests an inevitability to Conan Doyle’s success as well. The article commences with a portrait of the adult author buttoned up in his jacket, tie at his throat, by George Hutchinson (see figure 4), and this is facing the first page of the narrative; the author’s head is turned towards the text. Underneath is a facsimile of Conan Doyle’s signature: ‘yours very truly A Conan Doyle’ lending his account authority and adding to the impression of intimacy that we have seen fostered in the *Idler* (632). Then, on the first page of the narrative, facing in the opposite direction is a smaller illustration, this time by Sydney Cowell: ‘I was six’ (see figure 4). Cowell depicts the young Conan Doyle in the act of writing and the author appears little different from his adult image: similar dress, similar hair. Visually, there is a direct connection between the two illustrations, implying a connection between his childhood and his position as a popular author. His personal, passionate learning from an early age has led, through determined practice, to the expert, authoritative writer George Hutchinson depicts. Thus ‘My First Book’ suggests that Conan Doyle’s authority is deserved. This self-fashioning will inflect his medical stories in later issues of the *Idler* that explore authority and how it may be earned in a medical context.

## The Case of Lady Sannox

Like ‘The Los Amigos Fiasco’, ’The Case of Lady Sannox’ (Conan Doyle, 1894a) proposes to the readers of the *Idler* that at times the layperson is more competent than those with undeserved authority. This time though he makes the reader an active participant in that proposition and goes further to suggest that the skills associated with medical expertise are wasted if not brought to bear on wider experience. The story also suggests that morality has a significant impact on whether authority is deserved or not.

**Figure 5:**
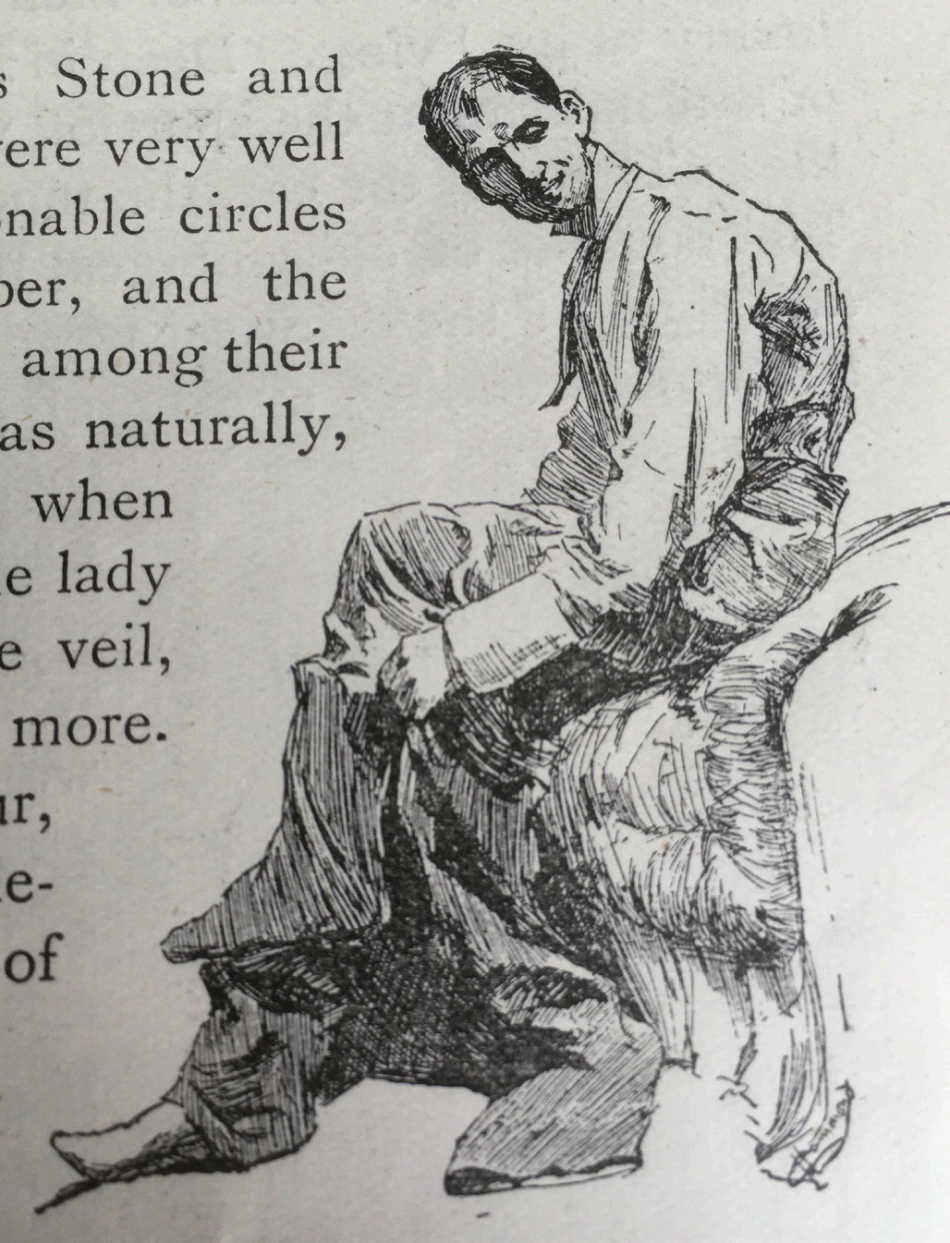
Misses Hammond. ‘Smiling pleasantly upon the universe’ (adapted from Conan Doyle, 1894a, 331).

He commences with a highly suggestive first scene that depicts the end of the story and is emphasised by the illustration ‘Smiling pleasantly upon the universe’ (see figure 5): the brilliant doctor has been left entirely without authority, 'smiling pleasantly upon the universe, with both legs jammed into one side of his breeches, and his great brain about as valuable as a cup full of porridge’ (331). Conan Doyle contrasts the scale between the vast compass of the Doctor’s gaze and the meagre capacity of his brain’s value, alongside the lopsidedness of his immobilising dressing, and thus presents Doctor Stone as entirely incapacitated, at odds with himself. The illustration uses a (perhaps accidental) awkward scale to portray the doctor: his head appears too small when compared with his legs, while his crumpled shirt somewhat swamps his bent body. Here is a man whose arrogant failure to use his expertise has debilitated his mind. Conan Doyle reveals that Stone broke the moral code: ‘two challenging glances and a whispered word’ and he commences an affair with a married woman—the Lady Sannox of the title (332). Clearly Conan Doyle intends us to connect two incidences of disorder. His narrative then follows Dr Stone until he finds himself gruesomely disfiguring his mistress in a horrific scene. He receives a mysterious visitor, who turns out to be the cheated husband, but Stone’s diagnostic skills fail him as he is deceived by the disguise in his hurry to oblige his visitor in order to earn an excessive sum of money quickly before he makes an illicit visit with his mistress. Haste and misreading mean he encounters (but fails to recognise) Lady Sannox before he expects to and the results not only disfigure his mistress, but also his mind.

Doctor Douglas Stone is highly skilled yet an incompetent reader; he fails to take his analytical skills outside of the medical sphere and employ them more widely. In contrast, Conan Doyle clearly intends his readers to decipher his clues as he builds our expectations; the evidence suggests something terrible awaits. The clues Conan Doyle places throughout the text serve to provide the reader with a more complex message than one of simple morality. Doctor Stone exhibits an arrogance that results in misdiagnosis and his failure to read what is clearly evident to the reader causes the tragic ending. Conan Doyle makes his clues readable in such a way as to position the reader as superior to the Doctor. He raises our suspicions as the narrative focuses on the episode with the mysterious visitor demanding his wife have part of her lip removed; in such a short fiction it must relate to the affair with Lady Sannox. Consequently we read the clues as pointing to the horror to come. The merchant visitor offers Stone an ‘extraordinarily high fee’ and yet takes him to a ‘mean-looking house’ the interior of which causes the doctor to ‘glanc[e] about him in some surprise’ at the lack of furniture or even carpet (335, 338). Many things seem wrong with this set-up: the woman is hidden; the merchant insists that no anaesthetic is used; and he says to Stone, ‘I can understand that the mouth will not be a pretty one to kiss’ (339). When Stone ‘grasp[s] the wounded lip with his forceps’, the reader recognises, and potentially recoils from, what he fails to see: he is about to disfigure his lover (339). Not only is Doctor Stone’s authority undeserved, the arrogance that accompanies it undoes him. When read alongside the other contributions Conan Doyle makes to the *Idler*, this story demonstrates that authority is something to be earned, but something that depends on more than just professional knowledge.

## The Doctors of Hoyland

‘The Doctors of Hoyland’ (Conan Doyle, 1894b), the final single issue piece Conan Doyle produced for the *Idler,* returns to some of the themes of previous pieces; he demonstrates the folly of assumptions about recognising authority, and suggests that it should be conferred by expertise. At the same time, he is concerned with the impact of change on authority, addressing the question of what happens when expert knowledge is disseminated in such a way as to expand a profession, in this case the slow trickle of women into medical practice.

Dr James Ripley visits a new doctor, Dr Verrinder Smith, who encroached on his territory by setting up a practice in his village. As with ‘The Case of Lady Sannox’, Conan Doyle writes clues for the reader, in this case clues to this interloper’s identity as Ripley sees them: first, the hall of the new doctor’s home (which also incorporates a consulting room) contains ‘two or three parasols and a lady’s sun bonnet’; second, the woman who greets him ‘held a pince-nez in her left hand’ as if she has just left off reading (229-30). Conan Doyle has immediately preceded this with a detailed description of the new doctor’s books; he builds his clues carefully. And yet when this woman announces that she is the doctor he seeks, ‘Dr Ripley was so surprised that he dropped his hat and forgot to pick it up again’ (230). In this case, it is less arrogance than simple assumption—a failure to attend to change in one’s own area of expertise. Ripley expected a man. Conan Doyle shows Ripley’s utter disappointment as leading him to interrupt his strict adherence to social codes: he behaves rudely and resists addressing the other doctor correctly. Indeed, Conan Doyle says that Ripley ‘felt that he had come very badly out of it’ (232). And he has; his response is a failure to convey all the expected signs of authority in his profession. Conan Doyle portrays the new doctor as superior to Ripley, not only in her social behaviour but also in her medical practice, for example she cures patients of ailments that had previously seemed unremitting or hopeless. Like Stulpnagel and Conan Doyle himself, she develops her expertise through practice; she reads the *Lancet* and has more up-to-date pamphlets than Ripley (231). Her authority is conferred by her adoption of conventional professional behaviour and her application of new knowledge. Expertise must necessarily move with the times.

## Conclusion

I began this essay with the questions raised by Conan Doyle’s character sketch of Koch as one of three competing narratives of medical science entangled in the *Review of Reviews* for the reader to evaluate. These questions were concerns related to the professionalisation of medicine and the relationships between the medical expert and the layman in the 1890s, where general practitioners depended on affecting an appearance of authority recognisable to their patients, and where patients, even though they had access to a proliferating popular medical press, were still susceptible to quackery. Known for his medical expertise, Conan Doyle’s illustrated work in the *Idler* bridges the gap between professional and layman as he writes in a publication that created an atmosphere of intimacy between its writers and readers, producing narratives that encourage their consideration of those questions.

Conan Doyle’s work invites his readers to consider the question of how knowledge is constructed. Both ‘De Profundis’ and ‘Glamour’ emphasise that competing narratives afford uncertainty, but that instability is part of the romance of being on the brink of the unknown. Imagination and mystery are difficult to resist and expertise does not negate them. Knowledge in these texts seems to involve holding these narratives together rather than conceiving of them as in competition. When it comes to the dissemination of knowledge, expertise trumps authority for Conan Doyle in the *Idler*, and if authority (understood as the power to influence) is pompous and laughable, then he suggests we should indeed laugh. His own authoritative influence is clearly connected to his hard-earned expertise, yet laughter is important again as a way to evade arrogance. This emphasis on expertise offers his readers a way to approach medical practitioners that might change the doctor/patient relationship from the deceptive one advised in Cathell’s guidance to doctors, to something more useful for the patient. Not that his writing in the *Idler* suggests character is unimportant; Dr Stone’s downfall implies that morality is a significant indicator of reliable medical authority and Dr Verrinder Smith conforms to expected professional behaviour. Most of all, Conan Doyle’s *Idler* narratives encourage the reader to have confidence in their judgements of the authority of their medical practitioners: the intimacy inherent in its periodical form (indicating the public are not so far removed from authority) and Conan Doyle’s careful placement of readable clues suggest his audience’s superiority over fallible medical practitioners. As the dissemination of medical knowledge was expanding within the medical profession, Conan Doyle’s writing connected in the *Idler* considers what it means to be a layman confronted with this evolving profession. He finds that the romance of discovery resists being consumed by expertise, but expertise is of the utmost importance and that the layperson can and should challenge unearned authority.

## Data Availability

No data are available.

## Notes

1. Count Mattei developed electrohomeopathic treatments, which although popular, were eventually largely dismissed as quackery.

2. Of the stories that will be discussed here, ‘The Case of Lady Sannox’ informs that reading, offering an example of the ‘ethical temptations’ faced by specialists rather than general practitioners (Kerr 2013, 63–4).

3. This conception of periodicals draws on Tattersdill (2016) discussion in his introduction to *Science, Fiction, and the Periodical Press*.

4. This essay does not provide a reading of ‘Sweethearts’. While that story does portray a doctor who can read medical symptoms but not the human heart, it does little more than suggest that more life experience would have benefited the doctor.

5. Tye (1974, 7) gives the sales of its first issue as 200 000 and adds that between 1895 and 1997 100 000 copies were produced per month.
